# Amino Acid Profiling Study of *Psidium guajava* L. Leaves as an Effective Treatment for Type 2 Diabetic Rats

**DOI:** 10.1155/2020/9784382

**Published:** 2020-04-23

**Authors:** Chang Xu, Xin Li, Debin Zeng, Ying Liu, Yuhang Gao, Makoto Tsunoda, Shiming Deng, Xi Xie, Rong Wang, Lu-shuang Li, Yanting Song, Yingxia Zhang

**Affiliations:** ^1^Key Laboratory of Tropical Biological Resources of Ministry of Education, Department of Pharmaceutical Sciences, School of Life and Pharmaceutical Sciences, Hainan University, Haikou 570228, China; ^2^Anning Hospital of Hainan Province, Haikou 570206, China; ^3^Research Center for Drug Safety Evaluation of Hainan Province, Haikou 571199, China; ^4^Graduate School of Pharmaceutical Sciences, The University of Tokyo, 7-3-1 Hongo, Bunkyoku, Tokyo 113-0033, Japan

## Abstract

Type 2 diabetes mellitus (T2DM) has become a major disease threatening human health worldwide. At present, the treatment of T2DM cannot cure diabetes and is prone to many side effects. *Psidium guajava* L. leaves have been reported to possess hypoglycemic activity, and they have been widely used in diabetes treatment in the folk. However, the antidiabetic mechanism has not been clearly explained. Also, the change in amino acid profile can reflect a metabolic disorder and provide insights into system-wide changes in response to physiological challenges or disease processes. The study found that *P. guajava* L. leaves can decrease fasting blood glucose and lipid levels in type 2 diabetic rats induced by streptozotocin. Through the analysis of amino acid profiling following 20 days of gavage administration, the concentration data were modeled by principal component analysis and orthogonal partial least squares discriminant analysis to find the different metabolites and related metabolic pathways (including cysteine and methionine metabolism, valine, leucine, and isoleucine biosynthesis, phenylalanine, tyrosine, and tryptophan biosynthesis) for the explanation of the hypoglycemic mechanism of *P. guajava* L., which provides an experimental and theoretical basis for diabetes prediction and for the development of new drugs for the treatment of diabetes.

## 1. Introduction

Diabetes mellitus (DM) is a chronic disease involving metabolic disorder with the characterization of long-term hyperglycemia. The number of diabetic patients has been increasing around the world in recent years [1]. There are more than 382 million people worldwide suffering from diabetes currently, and this figure has been projected to increase to 592 million by 2035 [2]. There are three main types of diabetes mellitus, namely, type 1 DM (T1DM), type 2 DM (T2DM), and gestational diabetes. In particular, cases of T2DM have been rising compared with cases of T1DM, an autoimmune disease that causes the destruction of islet beta cells and the inability of producing insulin. Hyperglycemia, insulin resistance, and obesity are common features in T2DM. The coexistence of these diseases is typically a case of metabolic syndrome, leading to possible cardiovascular disease [3, 4].

*Psidium guajava* L. (Myrtaceae) is native to tropical areas, such as the Caribbean, Central America, and South America [5]. *P. guajava* L. is highly regarded as a medical plant all over the world. Some earlier studies have reported on the effects of *P. guajava* L. for the treatment of gastroenteritis, dysentery, and diarrhea, and leaf extracts have also been reported to show biological activities, including antidiarrheal, antimicrobial, antioxidant, anti-inflammatory, antiallergy, antiplasmodial, antispasmodic, antidiabetic, antinociceptive, and antitussive activities [6, 7]. *P. guajava* L. leaves are widely used to treat DM and hypertension in South Africa [8] besides oriental countries [9, 10]. Additionally, researchers often pay attention to *P. guajava* L. leaves as a folk remedy for diabetes. Pharmacological compounds of *P. guajava* L. leaves contain tannins [11], flavonoids [12], carotenes [13], and various terpenoids [14, 15].

Amino acids are basic units that consist of protein, and their normal metabolism is one of the important foundations of life activities. Systemic histiocytes undergo amino acid metabolism and participate in protein synthesis through deamination, decarboxylation, ammonia metabolism, oxidative decomposition, etc. Amino acid metabolism occurs mainly through the liver, kidneys, and muscles. These tissues and organs all have an important influence on the amino acid metabolism library in the body. Defects in proteins and enzymes involved in amino acid metabolism, including some pathological conditions, result in changes in amino acid metabolism and serum amino acid profiles. The pathogenesis of diseases and the mechanism of action of drugs can be predicted, diagnosed, and studied by measuring the concentration of amino acids in human body fluids such as plasma, urine, and sweat [16–18]. Amino acid metabolic profiling can also be used to study the mechanism of T2DM, and potential diagnostic models were evaluated by characteristic curve analysis. Five amino acids (lysine, aspartic acid, threonine, methionine, and alanine) and two lipids (low-density lipoprotein cholesterol and high-density lipoprotein cholesterol) were found to be potential biomarkers of T2DM [19].

The aim of this study was to investigate the antihyperglycemic and antihyperlipidemic effects of *P. guajava* L. leaf extracts in T2DM rats. Through analyzing the changes in plasma amino acid profiles of type 2 diabetic rats treated by *P. guajava* L. leaf extracts and the correlation analysis of pharmacodynamics results, the pathway and the molecular mechanism of the hypoglycemic activity of *P. guajava* L. leaf extracts for type 2 diabetes were discussed. Therefore, we can provide a reference for the treatment of diabetes.

## 2. Materials and Methods

### 2.1. Reagents

Streptozotocin, amino acid standard solution (AAS18), and 6-aminocaproic acid were provided by Sigma-Aldrich (St. Louis, MO, USA). Citric acid monohydrate and trisodium citrate dihydrate were produced by Xilong Scientific Co., Ltd. (Guangdong, China). Glipizide tablets were supplied by Haikou Qili Pharmaceutical Co., Ltd. (Hainan, China). Chloral hydrate was obtained from Sinopharm Chemical Reagent Co., Ltd. (Shanghai, China). 4-Fluoro-7-nitro-2,1,3-benzoxadiazole (NBD-F) was provided by Tokyo Chemical Industry Co., Ltd. (Tokyo, Japan). Methanol (HPLC-grade) and acetonitrile (HPLC-grade) were purchased from Fisher Scientific, Ltd. (Rathburn, Walkerburn, UK). Trifluoroacetic acid (TFA), hydrochloric acid, and sodium hydroxide were obtained from Merida Technology Co., Ltd. (Beijing, China). Water was obtained from a Milli-Q water purification system (Bedford, USA). All other reagents were of analytical grade. All the biochemical analysis kits were purchased from Nanjing Jiancheng Bioengineering Institute.

### 2.2. Preparation of *P. guajava* L. Leaf Extracts

The *P. guajava* L. leaves were collected from Haidian Island (Haikou, China) on November 7, 2017. After collection, the plant was taxonomically identified and authenticated by Professor Yingxia Zhang, and voucher specimens (No. HDYX045) were deposited in the Herbarium of Medicinal Plants, Department of Pharmaceutical Sciences, School of Life and Pharmaceutical Sciences, Hainan University. The freshly collected *P. guajava* L. leaves were shade-dried. The dried *P. guajava* L. leaves were crushed with a stirrer, weighed, and mixed with 5 times the amount of distilled water, and then, extracts were leached at 60°C for 48 hours. The extracts were filtered by 20 layers of gauze, and the leaves were repeatedly extracted three times. The three filtrates were collected and filtered by a G3 glass sand funnel. The filtrate was concentrated in a 60°C water bath; then, the concentrated viscous extracts were dried in a 60°C oven; the dried *P. guajava* L. leaf extracts (GLE) were ground into powder, packed in a brown bottle, and placed in a dry box.

### 2.3. LC-MS/MS Analysis of *P. guajava* L. Leaf Extracts

A Thermo Ultimate 3000 UHPLC (Waltham, MA, USA) was used for this method. The chromatographic separations were carried out using a waters BEH C18 (2.1 × 150 mm, 1.7 *μ*m) column at 45°C, with a mobile phase composed of water containing 0.1% acetic acid (*A*) and acetonitrile containing 0.1% formic acid (*B*). The gradient elution condition was as follows: 5% *B* (0-1 min), 5%–85% *B* (1–23.5 min), 85%–96% *B* (23.5–24 min), and 96% *B* (24–26 min). The flow rate was set at 0.4 mL·min^−1^ and the injection volume was 10 *μ*L.

A Thermo Q-Exactive plus High-Resolution UHPLC/MS system (Q-TOF X500R, AB Sciex, Foster City, CA, USA) equipped with a heat electrospray ionization (HESI) was used for this analysis. Q-Exactive plus HRMS was scanned with the mass ranges *m*/*z* 70–1050 in both positive and negative ion modes. Spray voltage was set as +3.8 kV in positive ion mode and −3.0 kV in negative ion mode, respectively. The other conditions were illustrated as follows: capillary temperature: 320°C; sheath gas flow rate: 35 arb; aux gas heater temperature: 350°C; aux gas flow rate: 8 arb; S-lens RF level: 50 V; full ms resolution: 70000; MS/MS resolution: 17500; TopN: 5; NCE/stepped NCE: 20, 40.

### 2.4. Animals

Male SD rats were purchased from the Guangdong Medical Laboratory Animal Center (Guangdong, China). The rats were kept and maintained under laboratory conditions of temperature and light at 24°C and 12 h light/dark cycle, respectively. The experiment and the procedures were approved by the Animal Ethics Committee of Research Center for Drug Safety Evaluation of Hainan Province.

After one month of a high glucose and high fat diet, experimental diabetes was induced by an intraperitoneal injection of streptozotocin at 60 mg/kg body weight (STZ from Sigma, Beijing, China), dissolved in freshly prepared 0.1 M citrate buffer (pH of 4.0) in overnight fasted rats. The controls were treated with equivalent citrate buffer after one month of a standard diet. Five to seven days later, when the condition of diabetes was stabilized, animals were fasted for 8 h; then blood samples were taken from their tail veins and the plasma glucose concentration was measured by a blood glucose meter. Rats with blood glucose concentrations above 11.1 mmol/L and below 33.3 mmol/L were selected for the study.

### 2.5. Biochemical Analysis

One hour after the intragastric administration on the 18th day, each group of rats was given a glucose solution at 2 g/kg body weight. Blood samples were taken from tail veins at 0, 0.5, 1, and 2 hours in order to measure the blood sugar of rats in each group. The area under the blood sugar curve of the rats in each group was calculated according to the blood sugar values at four time points by using the following equation:

(1) $$\begin{array}{c} \text{AUC}\left(\text{mmol}\cdot \text{h}/\text{L}\right)=\left(A+B\right)\times 0.25+\left(B+C\right)\times 0.25+\left(C+D\right)\times 0.5, \end{array}$$

where *A*, *B*, *C*, and *D* represent blood glucose levels at 0, 30, 60, and 120 min, respectively.

After 20 days of gavage, the rats were anesthetized with 10% chloral hydrate solution at 3.5 mL/kg. The abdominal aortic blood was dissected to obtain 3–4 mL, and the serum was separated by centrifugation at 4500 r/min for 20 minutes. The glycosylated serum protein (GSP) content was determined by the fructose amine method, the total cholesterol (TCH) was determined by the cholesterol oxidase-peroxide coupled enzyme (COD-PAP) method, and the triglyceride (TG) content was determined by the glycerol phosphate oxidase-peroxide coupled enzyme (GPO-PAP) method. Red blood cells were used to prepare lysed blood, and the contents of glycated hemoglobin (GHb), high-density lipoprotein cholesterol (HDL-C), and low-density lipoprotein cholesterol (LDL-C) were determined by the kit.

### 2.6. Experimental Design

*P. guajava* L. leaf extracts, glipizide, and distilled water were administered orally to experimental rats during the course of 20 days. Animals were randomly divided into four equal groups of 4 rats each as follows.

- Healthy control group: normal rats were given distilled water orally.
- Model group: diabetic rats were given distilled water orally.
- Positive control group: diabetic rats were given glipizide daily at a dose of 5 mg/kg body weight.
- GLE-treated group: diabetic rats were supplemented daily with *P. guajava* L. leaf extracts at a dose of 800 mg/kg body weight.

At 20 days, rats were measured for their blood sugar, taking the blood of the tail vein after intragastric gavage, following fasting for 2 h. Plasma was isolated for amino acid analysis. Each sample was analyzed in triplicate.

A fluorescence derivatization procedure and pretreatment of rat plasma samples were performed according to our previous reports [20]. Approximately 500 *μ*L of the SD rat's blood was placed in an anticoagulant tube, kept in a refrigerator at 4°C for 30 min, and then centrifuged at 8000 r/min for 15 min. The supernatant was taken and stored frozen at −80°C until analysis. Forty microliters of plasma sample and 40 *μ*L of an internal standard solution (0.1 mM 6-aminocaproic acid) were added to 160 *μ*L of methanol, 160 *μ*L of acetonitrile. After the mixture was centrifuged at 5000 r/min for 5 min, 20 *μ*L of the supernatant was evaporated to dryness under reduced pressure for 1 h. Then, the residue was added to 180 *μ*L of 0.2 M borate buffer solution (pH 8.5). 40 *μ*L of 10 mM NBD-F was added and the mixture was heated in a water bath at a temperature of 60°C for 5 min. After cooling the reaction mixture in ice water, 240 *μ*L of a 100 mM HCl solution was added to the reaction mixture. The resultant solution was injected into the HPLC system. An HPLC system equipped with a Waters 1525 pump and 2489 detector (Milford, USA) was utilized. The separation of amino acids was carried out on an Agilent Poroshell HPH-C18 column (3 × 150 mm, 2.7 *μ*m). The flow rate was maintained at 0.4 mL/min, with UV detection at 472 nm [21]. The column temperature was kept at 30°C. The injection amount was 5 *μ*L. Mobile phase *A* was H_2_O/CH_3_CN/TFA (90/10/0.12, v/v/v), and mobile phase *B* was H_2_O/CH_3_CN/TFA (10/90/0.12, v/v/v). The gradient elution program of the mobile phases was shown as follows: 100% (*A*) from 0 to 10.5 min, 100–70% (*A*) from 10.5 to 12.0 min, 70% (*A*) from 12.0 to 15.0 min, 70–58% (*A*) from 15.0 to 22.0 min, 58–0% (*A*) from 22.0 to 23.0 min, 0% (*A*) from 23.0 to 24.0 min, and 0–100% (*A*) from 24.0 to 24.1 min.

## 3. Results and Discussion

### 3.1. Analysis of Constituents in *P. guajava* L. Leaf Extracts

**Figure 1:**
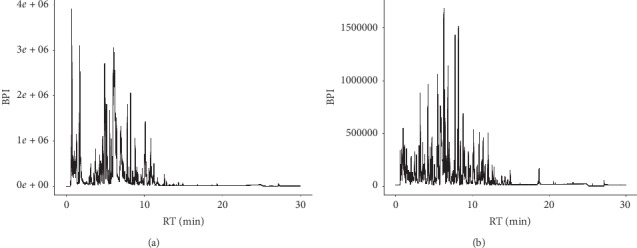
The basic peak chromatograms (BPCs) of *P guajava* L. leaf extracts in the negative ion mode (a) and in the positive ion mode (b).

*P. guajava* L. leaf extracts were analyzed with UHPLC-Q-TOF-MS/MS in both positive and negative ion modes. The representative basic peak chromatograms (BPCs) corresponding to positive and negative signals of *P. guajava* L. leaf extracts are shown in Figure 1. The retention time, molecular weights, and fragment ions of identified compounds are listed in Table 1. Finally, a total of 26 constituents were identified (Table 1).

### 3.2. Biomedical Results

**Figure 2:**
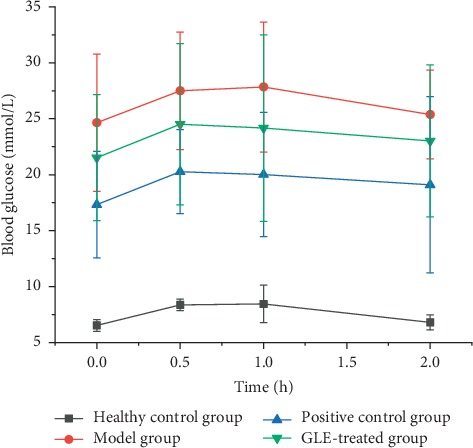
Effect of *P. guajava* L. leaves on glucose tolerance in type 2 diabetic rats.

The biomedical characteristics of healthy control, model, positive control, and GLE-treated groups including body weight, glycosylated hemoglobin, serum insulin, serum triglycerides, serum total cholesterol, HDC-C, LDL-C, glycosylated serum protein, and glucose tolerance were determined. The fasting plasma glucose of the GLE-treated group (21.67 ± 5.67 mmol/L) was lower than that of the model group (Table 2 and Figure 2). Besides, the ANCOVA analysis showed that total cholesterol, LDL-C, and glycosylated serum protein in the GLE-treated group were significantly reduced as compared with the model group (*p* < 0.05). These results indicated that GLE possesses antihyperglycemic and antihyperlipidemic effects.

### 3.3. Multivariate Statistical Analysis

**Figure 3:**
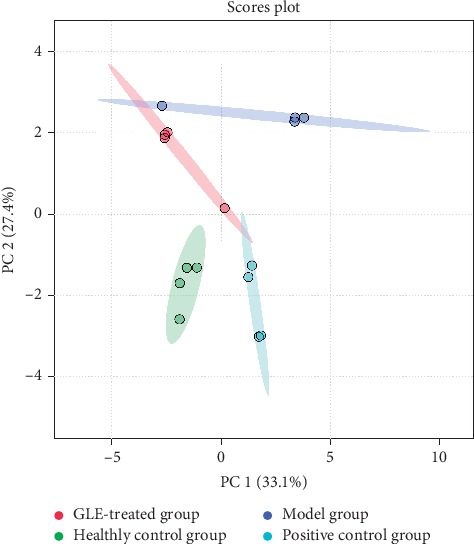
Principal component analysis (PCA) score plot based on the amino acid profiling of the rat plasma samples collected from the healthy control (green), model (dark blue), positive control (light blue), and GLE-treated groups (red).

Amino acids in rat plasma samples were separated and determined by HPLC. The concentrations of amino acids in the rat plasma samples were analyzed (Table 3). The data were normalized with MetaboAnalyst 4.0, and then principal component analysis (PCA) was performed to obtain the score vectorgraph of amino acid profiles of rat plasma in each group. The similarities and intergroup differences between the plasma samples of the four groups of rats were observed preliminarily through the established PCA model. The differences between samples were maximized by the PCA model (Figure 3), while it is not possible to distinguish whether the differences originate from intergroup or intragroup relationships.

**Figure 4:**
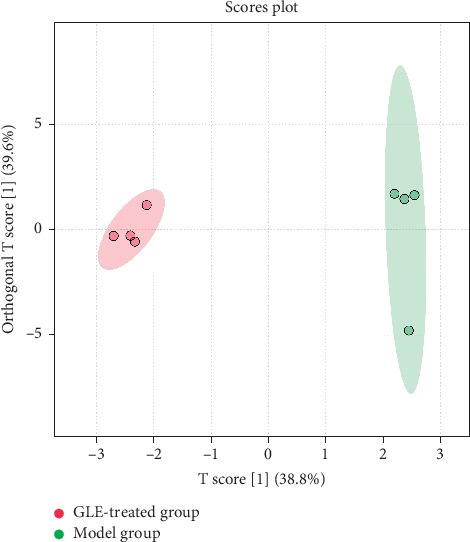
Orthogonal partial least squares (OPLS) score plot of amino acid profiling in the rat plasma samples collected from the model (green) and GLE-treated (red) groups.

The orthogonal partial least squares discriminant analysis (OPLS-DA) model was established to fully extract the differences between groups. Before the OPLS-DA model is used for analysis, it is necessary to set up the actual sample groups and perform a general analysis for two groups, in order to enhance the interpretation ability and classification performance of the model. Therefore, the OPLS-DA models of the model group and the drug-administered groups were established (Figure 4). The aggregation of groups and the dispersion of intergroup differences were increased by comparing with the PCA model, indicating that the model group and the drug-administered groups can be better distinguished by using the OPLS-DA model.

### 3.4. Screening of Pharmacological Markers

In order to find potential amino acid biomarkers for the treatment of T2DM with *P. guajava* L. leaf extracts, the amino acid (VIP >1) models were selected as characteristic metabolites, which were obtained by the OPLS-DA model (Table 4). VIP means the variable importance in projection (VIP) score. VIP was used for the calculation of the importance of variables in the model, and the overall contribution of each variable to the model is described, usually with a threshold of VIP >1.

**Figure 5:**
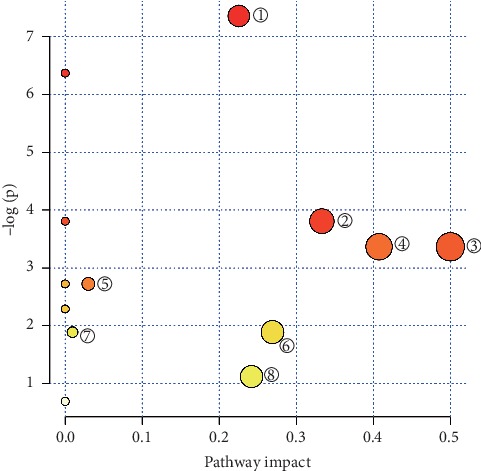
Effect of *P. guajava* L. leaf extracts on amino acid metabolic pathways in type 2 diabetes mellitus rats.

The characteristic metabolites were introduced into the MetaboAnalyst 4.0 database in order to analyze metabolic pathways and find related metabolic pathways. As can be seen in Figure 5, dots represent the corresponding metabolic pathways, and colors represent the *p* values of the metabolic pathways. The redder the color, the larger the −log(*p*); in other words, P is smaller. The radius of the dot represented the Impact value of the metabolic pathway: the larger the radius, the greater the Impact. As indicated in Figure 4 and Table 5, the administration of *P. guajava* L. leaf extracts was associated with metabolic pathways in T2DM rats, such as cysteine and methionine metabolism, valine, leucine, and isoleucine biosynthesis, and phenylalanine, tyrosine, and tryptophan biosynthesis. The Impact values of cysteine and methionine metabolism, valine, leucine, and isoleucine biosynthesis, phenylalanine metabolism, glycine, serine, and threonine metabolism, and histidine metabolism were greater than 0.10, which indicated that these amino acid metabolic pathways are significant for the hypoglycemic activity of *P. guajava* L. leaf extracts.

## 4. Conclusions

In this study, the blood glucose of T2DM rats decreased after they were treated with *P. guajava* L. leaf extracts. Through the analysis of amino acid profiling following 20 days of gavage administration, the concentration data were analyzed by principal component analysis and orthogonal partial least squares discriminant analysis to find the biomarkers and related metabolic pathways. Among these metabolic pathways, cysteine and methionine metabolism, valine, leucine, and isoleucine biosynthesis, phenylalanine metabolism, glycine, serine, and threonine metabolism, and histidine metabolism were found to be significant for the hypoglycemic activity of *P. guajava* L. leaf extracts.

## Figures and Tables

**Table 1:** Identification of the chemical compounds of *P guajava* L. leaf extracts by UPLC-Q-TOF-MS/MS.

Identification of the chemical compounds of *P. guajava* L. leaf extracts in negative ion mode				
Identification	RT (min)	[M − H]^−^ (*m*/*z*)	Molecular formula	Fragment ions (*m*/*z*)^*a*^

Malic acid	0.839	133.014	C_4_H_6_O_5_	115, 89, 72, 71, 59
Gallocatechin	2.891	305.065	C_15_H_14_O_7_	219, 167, 165, 137, 125
Gossypetin	2.895	479.086	C_21_H_20_O_13_	319, 275, 225, 203, 110
3,4-Dihydroxybenzoic acid	4.365	153.019	C_7_H_6_O_4_	109, 108
4-Hydroxybenzoic acid	4.423	137.024	C_7_H_6_O_3_	108, 93, 65
Coumaric acid	4.620	163.040	C_9_H_8_O_3_	119
Rutin	6.186	609.149	C_27_H_30_O_16_	285, 284, 255
Isoquercitrin	6.447	463.087	C_21_H_20_O_12_	301, 300, 271, 255
Avicularin	6.927	433.076	C_20_H_18_O_11_	301, 300, 271, 255, 243
Kaempferol-3-*O*-glucoside	7.104	447.095	C_21_H_20_O_11_	284, 255, 227
Myricetin	7.560	317.030	C_15_H_10_O_8_	178, 151, 137, 109
Kaempferol	10.175	285.040	C_15_H_10_O_6_	165, 153, 121

Identification of the chemical compounds of *P. guajava* L. leaf extracts in positive ion mode				
Identification	RT (min)	[M + H]^+^ (*m*/*z*)	Molecular formula	Fragment ions (*m*/*z*)^*a*^
Ellagic acid	0.904	303.013	C_14_H_6_O_8_	285, 275, 257, 201
Gallic acid	1.180	171.028	C_7_H_6_O_5_	107, 81, 79, 53, 51
Epicatechin	3.553	291.085	C_15_H_14_O_6_	203, 137, 125, 123, 109
Catechin	4.449	291.086	C_15_H_14_O_6_	147, 139, 123
Genistein	4.585	271.060	C_15_H_10_O_5_	215, 153, 149, 141
Procyanidin B2	4.894	579.149	C_30_H_26_O_12_	409, 287, 139, 127, 123
Chlorogenic acid	5.078	355.101	C_16_H_18_O_9_	193, 179, 123, 105, 77
Ferulic acid	6.025	195.064	C_10_H_10_O_4_	149, 145, 134, 117, 89
Quercetin-3-*O*-galactoside	6.294	465.101	C_21_H_20_O_12_	303, 229, 153, 85, 61
Quercetin	6.300	303.048	C_15_H_10_O_7_	257, 229, 153, 137
Tamarixetin	7.134	317.065	C_16_H_12_O_7_	302, 285, 229, 153
Caffeic acid	7.786	181.049	C_9_H_8_O_4_	145, 135, 117, 89, 63
Morin	8.817	303.048	C_15_H_10_O_7_	257, 229, 153, 137
Formononetin	13.998	269.080	C_16_H_12_O_4_	120, 105, 77, 53, 51

**Table 2:** Biomedical characteristics of healthy control, model, positive, and GLE-treated groups.

	Healthy control group	Model group	Positive control group	GLE-treated group
Body weight (g)	430.18 ± 22.37	262.77 ± 12.38^*a*^	366.16 ± 16.55^*ab*^	336.24 ± 23.27^*ab*^
Fasting blood glucose (mmol/L)	6.88 ± 0.36	24.80 ± 4.68^*a*^	18.63 ± 6.13^*ab*^	21.67 ± 5.67^*a*^
Triglyceride (mmol/L)	1.66 ± 0.77	2.84 ± 0.11^*a*^	2.61 ± 0.30^*a*^	2.79 ± 0.07^*a*^
Total cholesterol (mmol/L)	2.29 ± 0.09	4.80 ± 0.40^*a*^	2.38 ± 0.58^*b*^	4.05 ± 0.19^*ab*^
HDL cholesterol (mmol/L)	2.76 ± 0.76	1.75 ± 0.21^*a*^	1.77 ± 0.84^*a*^	2.00 ± 0.69^*ab*^
LDL cholesterol (mmol/L)	0.10 ± 0.56	0.66 ± 0.06^*a*^	0.31 ± 0.37^*ab*^	0.51 ± 0.68^*ab*^
Glycosylated hemoglobin (mmol/L)	13.23 ± 2.12	19.47 ± 4.33^*a*^	14.26 ± 4.23^*b*^	18.32 ± 2.35^*a*^
Glycosylated serum protein (mmol/L)	1.24 ± 0.14	1.79 ± 0.18^*a*^	1.36 ± 0.13^*b*^	1.47 ± 0.16^*ab*^

Values are means ± standard deviation number of rats. ^*a*^*p* value indicates that the difference between the current group and the healthy control group was less than 0.05. ^*b*^*p* value indicates that the difference between the current group and the model group was less than 0.05.

**Table 3:** The contents of amino acids in healthy control, model, positive, and GLE-treated groups.

	Healthy control group (*μ*mol/L) (*n* = 4)	Model group (*μ*mol/L) (*n* = 4)	Positive control group (*μ*mol/L) (*n* = 4)	GLE-treated group (*μ*mol/L) (*n* = 4)
His	37.9 ± 0.9	38.88 ± 0.67	31.19 ± 0.44	47.07 ± 1.06
Asn	34.7 ± 12.63	49.97 ± 6.51	27.99 ± 8.26	50.31 ± 8.87
Gln	428.66 ± 63.89	259.76 ± 13.07	179.44 ± 44.9	429.33 ± 128.92
Ser	104.74 ± 14.8	91.77 ± 2.12	67.43 ± 16.44	102.33 ± 12.36
Arg	48.87 ± 8.59	38.45 ± 18.3	43.95 ± 2.65	25.66 ± 5.15
Gly	280.25 ± 43.87	95.54 ± 19.69	205.3 ± 37.14	206.85 ± 6.99
Glu	44.53 ± 8.13	42.78 ± 33.24	26.2 ± 2.26	25.54 ± 2.19
Thr	143.33 ± 33.19	161.25 ± 28.71	97.71 ± 16.77	147.55 ± 34.94
Ala	408.44 ± 38.8	345.73 ± 96.12	261.21 ± 33.34	262.4 ± 30.53
Pro	89.65 ± 19.1	131.28 ± 49.69	72.98 ± 8.84	96.64 ± 9.44
Met	18.91 ± 1.19	38.53 ± 9.86	14.57 ± 2.76	21.16 ± 1.98
Val	89.71 ± 18.4	192.09 ± 79.9	139.47 ± 23.35	96.48 ± 32.92
Orn	11.27 ± 5.33	62.9 ± 32.42	12.56 ± 3.29	168.72 ± 68
Cys	52.72 ± 16.36	29.71 ± 1.15	48.84 ± 3.94	38.69 ± 2.76
Lys	162.16 ± 13.92	86.59 ± 5.12	66.88 ± 1.98	121.93 ± 32.86
Leu	87.18 ± 12.15	176.97 ± 73.47	137.26 ± 7	90.76 ± 18.1
Phe	44.46 ± 5.74	43.95 ± 1.52	48.94 ± 5.66	50.68 ± 4.52

**Table 4:** VIP contents in the orthogonal partial least squares discriminant analysis (OPLS-DA) model.

Amino acid	VIP
His	1.46
Cys	1.36
Phe	1.13
Gln	1.08
Val	0.99
Ser	0.86
Pro	0.73
Glu	0.55
Asn	0.05
Gly	1.45
Met	1.21
Orn	1.10
Leu	1.01
Lys	0.96
Ala	0.82
Arg	0.71
Thr	0.35

**Table 5:** Effect of *P. guajava* L. leaf extracts on amino acid metabolic pathways in T2DM rats.

No.	Pathway name	*p*	−log(*p*)	Holm *p*	FDR	Impact
1	Cysteine and methionine metabolism	6.4026*E *−* *4	7.3536	0.010884	0.010884	0.22557
2	Valine, leucine, and isoleucine biosynthesis	0.0222	3.8076	0.333	0.094351	0.33333
3	Phenylalanine, tyrosine, and tryptophan biosynthesis	0.034516	3.3663	0.44871	0.097795	0.5
4	Phenylalanine metabolism	0.034516	3.3663	0.44871	0.097795	0.40741
5	Primary bile acid biosynthesis	0.06557	2.7246	0.72127	0.11147	0.02976
6	Glycine, serine, and threonine metabolism	0.1508	1.8918	0.90481	0.1972	0.26884
7	Glutathione metabolism	0.1508	1.8918	0.90481	0.1972	0.00955
8	Histidine metabolism	0.32563	1.122	1.0	0.39541	0.24194

## Data Availability

The data used to support the findings of this study are available from the corresponding author upon request.
